# *WFS1* mutation screening in a large series of Japanese hearing loss patients: Massively parallel DNA sequencing-based analysis

**DOI:** 10.1371/journal.pone.0193359

**Published:** 2018-03-12

**Authors:** Masafumi Kobayashi, Maiko Miyagawa, Shin-ya Nishio, Hideaki Moteki, Taro Fujikawa, Kenji Ohyama, Hirofumi Sakaguchi, Ikuyo Miyanohara, Akiko Sugaya, Yasushi Naito, Shin-ya Morita, Yukihiko Kanda, Masahiro Takahashi, Kotaro Ishikawa, Yuki Nagano, Tetsuya Tono, Chie Oshikawa, Chiharu Kihara, Haruo Takahashi, Yoshihiro Noguchi, Shin-ichi Usami

**Affiliations:** 1 Department of Otorhinolaryngology, Shinshu University School of Medicine, Matsumoto, Japan; 2 Department of Hearing Implant Sciences, Shinshu University School of Medicine, Matsumoto, Japan; 3 Department of Otolaryngology, Tokyo Medical and Dental University, Tokyo, Japan; 4 Department of Otorhinolaryngology, Tohoku Rosai Hospital, Sendai, Japan; 5 Department of Otorhinolaryngology-Head and Neck Surgery, Kyoto Prefectural University of Medicine, Kyoto, Japan; 6 Department of Otolaryngology-Head and Neck Surgery, Kagoshima University Graduate School of Medical and Dental Sciences, Kagoshima, Japan; 7 Department of Otolaryngology—Head and Neck Surgery, Okayama University Graduate School of Medicine, Dentistry and Pharmaceutical Sciences, Okayama, Japan; 8 Department of Otolaryngology, Kobe City Medical Center, Kobe, Japan; 9 Department of Otolaryngology-Head and Neck Surgery, Hokkaido University Graduate School of Medicine, Sapporo, Japan; 10 Kanda ENT Clinic, Nagasaki Bell Hearing Center, Nagasaki, Japan; 11 Department of Otorhinolaryngology-Head and Neck Surgery, Yokohama City University School of Medicine, Yokohama, Japan; 12 Department of Otolaryngology, National Rehabilitation Center for Persons with Disabilities, Tokorozawa, Saitama, Japan; 13 Department of Otolaryngology, Faculty of Medicine, University of Miyazaki, Miyazaki, Japan; 14 Department of Otorhinolaryngology, Graduate School of Medical Sciences, Kyushu University, Fukuoka, Japan; 15 Department of Otolaryngology-Head and Neck Surgery, Nagasaki University Graduate School of Biomedical Sciences, Nagasaki, Japan; Sant Joan de Déu Children's Hospital, SPAIN

## Abstract

A heterozygous mutation in the Wolfram syndrome type 1 gene (*WFS1*) causes autosomal dominant nonsyndromic hereditary hearing loss, DFNA6/14/38, or Wolfram-like syndrome. To date, more than 40 different mutations have been reported to be responsible for DFNA6/14/38. In the present study, *WFS1* variants were screened in a large series of Japanese hearing loss (HL) patients to clarify the prevalence and clinical characteristics of DFNA6/14/38 and Wolfram-like syndrome. Massively parallel DNA sequencing of 68 target genes was performed in 2,549 unrelated Japanese HL patients to identify genomic variations responsible for HL. The detailed clinical features in patients with *WFS1* variants were collected from medical charts and analyzed. We successfully identified 13 *WFS1* variants in 19 probands: eight of the 13 variants were previously reported mutations, including three mutations (p.A684V, p.K836N, and p.E864K) known to cause Wolfram-like syndrome, and five were novel mutations. Variants were detected in 15 probands (2.5%) in 602 families with presumably autosomal dominant or mitochondrial HL, and in four probands (0.7%) in 559 sporadic cases; however, no variants were detected in the other 1,388 probands with autosomal recessive or unknown family history. Among the 30 individuals possessing variants, marked variations were observed in the onset of HL as well as in the presence of progressive HL and tinnitus. Vestibular symptoms, which had been rarely reported, were present in 7 out of 30 (23%) of the affected individuals. The most prevalent audiometric configuration was low-frequency type; however, some individuals had high-frequency HL. Haplotype analysis in three mutations (p.A716T, p.K836T, and p.E864K) suggested that the mutations occurred at these mutation hot spots. The present study provided new insights into the audiovestibular phenotypes in patients with *WFS1* mutations.

## Introduction

Hearing loss (HL) is the most prevalent sensory impairment, and is reported to occur at a rate of 1.86 per 1000 newborns [1]. HL can be caused by environmental and/or hereditary factors. Hereditary HL accounts for 68% of congenital HL cases, and for 54% of HL at the age of four years [1]. In addition, 70% of hereditary HL cases show nonsyndromic HL, without any associated symptoms. The inheritance pattern of this nonsyndromic hereditary HL is divided into autosomal dominant, autosomal recessive, X-linked, and mitochondrial. Autosomal dominant nonsyndromic HL (ADNSHL) occurs in approximately 20% of nonsyndromic hereditary HL cases [2]. It is genetically heterogeneous, and 36 causative genes for ADNSHL have been identified to date [3].

Heterozygous mutations in the Wolfram syndrome type 1 gene (*WFS1*) are responsible for one form of ADNSHL, DFNA6/14/38 (MIM #600965) [4], and Wolfram-like syndrome (MIM #614296) [5–7]. *WFS1* was first identified as a causative gene for the autosomal recessive disorder Wolfram syndrome type 1 (MIM #222300), or DIDMOAD (diabetes insipidus, diabetes mellitus, optic atrophy, and deafness) syndrome [8, 9]. *WFS1* is located on chromosome 4p16.1, contains eight exons, and encodes wolframin, which is an 890-amino acid transmembrane protein. Biochemical studies suggest that wolframin is localized predominantly in the endoplasmic reticulum, and plays a role in membrane trafficking, protein processing and/or regulation of endoplasmic reticulum calcium homeostasis [10]. Immunohistochemical and in situ hybridization studies in the mouse inner ear revealed that wolframin is expressed in various cell types in the cochlea and vestibule from postnatal day 1 to 35, and it is thought to play a possible role in inner ear ion (K^+^ and/or Ca^2+^) homeostasis [11].

To date, over 40 different heterozygous mutations in *WFS1* have been reported to cause DFNA6/14/38, with the majority of the mutations located in exon 8 [12–15]. The audiometric configuration of DFNA6/14/38 patients is characterized by symmetrical, low-frequency sensorineural HL with or without progression. The onset of HL varies widely from prelingual to the early 30s. By contrast, vestibular impairments and/or vestibular function abnormalities are extremely rare associated symptoms among DFNA6/14/38 patients, and only one paper has reported affected individuals showing vertiginous attacks [16]. Some heterozygous mutations in *WFS1* can cause Wolfram-like syndrome, which is characterized by autosomal dominant inherited HL with optic atrophy and/or diabetes mellitus [5–7].

We previously screened for *WFS1* variants in 206 Japanese ADNSHL and 64 autosomal recessive nonsyndromic hereditary HL (ARNSHL) probands, and identified two previously reported mutations in three unrelated families with ADNSHL [17]. In the present study, we used massively parallel DNA sequencing (MPS) for the mutational analysis of the *WFS1* gene among a larger series of 2,549 unrelated Japanese hereditary HL patients. The aims of the study were to estimate the prevalence of *WFS1* mutations in the Japanese hereditary HL population, and provide a more precise description of the clinical features. Additionally, haplotype analysis was performed on three missense mutations identified in multiple families to confirm whether these mutations occurred in a mutational hotspot.

## Materials and methods

### Subjects

Sixty-seven otolaryngology departments across Japan participated in the present study, and a total of 2,549 unrelated Japanese patients (probands) with HL were enrolled. The inheritance pattern of HL in the probands’ families was assumed to be autosomal dominant or mitochondrial inheritance in 602 families, autosomal recessive in 1,018, sporadic in 559, and unknown in 370. Low-frequency sensorineural HL based on pure-tone audiograms as defined previously [18] was recognized in 55 of 602 probands with autosomal dominant HL, and in 67 of 1,577 probands with autosomal recessive or sporadic HL.

Written informed consent was obtained from all subjects (or from their next of kin, caretaker, or guardian in the case of minors/children) prior to enrollment in this study. All procedures were approved by the Shinshu University Ethical Committee as well as the respective Ethical Committees of the other participating institutions

### Mutational analysis

Amplicon libraries were prepared using an Ion AmpliSeq™ Custom Panel (Applied Biosystems, Life Technologies), in accordance with the manufacturer’s instructions, for 68 genes reported to cause non-syndromic hereditary HL (S1 Table). The detailed protocol has been described elsewhere [19]. After preparation, the amplicon libraries were diluted to 20pM and equal amounts of 6 libraries for 6 patients were pooled for one sequence reaction.

Emulsion PCR and sequencing were performed according to the manufacturer’s instructions. The detailed protocol has been described elsewhere [19]. MPS was performed with an Ion Torrent Personal Genome Machine (PGM) system using an Ion PGM™ 200 Sequencing Kit and an Ion 318™ Chip (Life Technologies).

The sequence data were mapped against the human genome sequence (build GRCh37/hg19) with a Torrent Mapping Alignment Program. After sequence mapping, the DNA variant regions were piled up with Torrent Variant Caller plug-in software. After variant detection, their effects were analyzed using ANNOVAR software [20, 21]. The missense, nonsense, insertion/deletion and splicing variants were selected from the identified variants. Variants were further selected as less than 1% of 1) the 1,000 genome database [22], 2) the 6,500 exome variants [23], 3) the Human Genetic Variation Database (dataset for 1,208 Japanese exome variants) [24], and 4) the 333 in-house Japanese normal hearing controls. Direct sequencing was conducted to confirm the selected variants.

The pathogenicity of a variant was evaluated by ACMG (American College of Medical Genetics) standards and guidelines [25]. For missense variants, in particular, functional prediction software, including Sorting Intolerant from Tolerant (SIFT) [26], Polymorphism Phenotyping (PolyPhen2) [27], LRT [28], Mutation Taster [29], Mutation Assessor [30], Functional Analysis through Hidden Markov Models (FATHMM) [31], RadialSVM, and LR [32], available on the wANNOVAR website were applied. Conservation of the mutation site was also evaluated in 13 species from the Homologene website [33]. Segregation analysis was performed for each proband and their family members.

### Haplotype analysis

Haplotype analysis was performed to obtain insights into the origin of three mutations (p.A716T, p.K836T, and p.E864K) that were detected in multiple families. A set of 36 single nucleotide polymorphisms (SNPs) within the 1-Mbp region linked to the *WFS1* locus (6271577–6304992; 17 sites upstream, 17 sites downstream, 2 sites in the *WFS1* gene) was analyzed using direct DNA sequencing. The mutation-linked haplotype was determined by family member segregation analysis and compared among unrelated families with the same mutations. With respect to the p.K836T missense mutation, a large Japanese family with the identical mutation in our previous report [34] was included in the comparison.

### Clinical evaluation

The onset age of HL as well as the presence of subjective progression in hearing, tinnitus, and vertigo/dizziness were analyzed based on the medical charts of the probands and their affected family members. Information on the incidence of diabetes mellitus and optic atrophy was also collected and evaluated.

Pure-tone audiometry or conditioned orientation response audiometry (COR) was performed to evaluate HL according to the tested age of each individual. The pure-tone average (PTA) was calculated from the audiometric thresholds at four frequencies (0.5, 1, 2, and 4 kHz). The HL patients were divided into 4 groups based on severity; mild (PTA: 20–40 dB HL), moderate (41–70 dB HL), severe (71–95 dB HL), and profound (>95 dB HL). The audiometric configurations were classified into low-frequency, middle-frequency (U-shaped), high-frequency, low- and high-frequency and flat types as reported previously [18].

## Results

### Detected variants

MPS detected eight previously reported mutations and five novel, possibly pathogenic variants in *WFS1* in 19 of the 2549 Japanese HL probands (Table 1). All heterozygous missense variants were identified within exon 8, and were confirmed by Sanger sequencing. Three mutations (p.A684V, p.K836N, and p.E864K) were previously reported to cause Wolfram-like syndrome [5–7]. No candidate pathogenic variants in the other 68 deafness genes were detected in the 19 probands.

**Table 1:** *WFS1* mutations found in this study.

				Prediction software*									
Nucleotide Change	Exon	Amino Acid Change	Domain	SIFT	PolyPhen2_HVIR	PolyPhen2_HVAR	LRT	Mut_Taster	Mut_Assessor	FATHMM	RadialSVM	LR	Allele frequency in 333 in-house controls
Previously reported mutations													
c.1846G>T	8	p.A616S	–	0.61	0.17	0.05	0.96	1.00	0.65	0.52	0.44	0.71	0
c.2051C>T	8	p.A684V	C-terminal	1.00	1.00	0.96	1.00	1.00	0.72	0.55	0.72	0.94	0
c.2146G>A	8	p.A716T	C-terminal	0.92	1.00	0.81	1.00	1.00	0.69	0.55	0.7	0.92	0
c.2185G>A	8	p.D729N	C-terminal	0.38	0.06	0.01	1.00	0.97	0.55	0.55	0.52	0.72	0
c.2385G>C	8	p.E795D	C-terminal	0.73	0.02	0.02	1.00	0.92	0.6	0.52	0.39	0.6	0
c.2507A>C	8	p.K836T	C-terminal	0.38	1.00	0.95	1.00	1.00	0.57	0.52	0.57	0.78	0
c.2508G>C	8	p.K836N	C-terminal	0.90	1.00	0.95	1.00	1.00	0.57	0.52	0.6	0.78	0
c.2590G>A	8	p.E864K	C-terminal	0.93	1.00	1.00	1.00	1.00	0.57	0.52	0.65	0.82	0
Novel mutations													
c.908T>C	8	p.L303P	N-terminal	1.00	1.00	1.00	1.00	1.00	0.65	0.55	0.36	0.31	0
c.923C>G	8	p.S308C	N-terminal	0.97	1.00	1.00	1.00	1.00	0.66	0.49	0.64	0.79	0
c.1982A>G	8	p.N661S	C-terminal	0.65	1.00	0.94	1.00	1.00	0.71	0.55	0.7	0.93	0
c.2027G>A	8	p.R676H	C-terminal	0.87	1.00	0.91	1.00	0,98	0.67	0.56	0.64	0.93	0
c.2045A>G	8	p.N682S	C-terminal	0.44	0.97	0.82	1.00	1.00	0.66	0.55	0.67	0.89	0

*The prediction scores of each algorithm included on the wANNOVAR website were converted from the original scoring system. Scores closer to 1.0 indicated the mutation was more damaging, and those closer to 0 indicated they were more tolerant.

**Fig 1:**
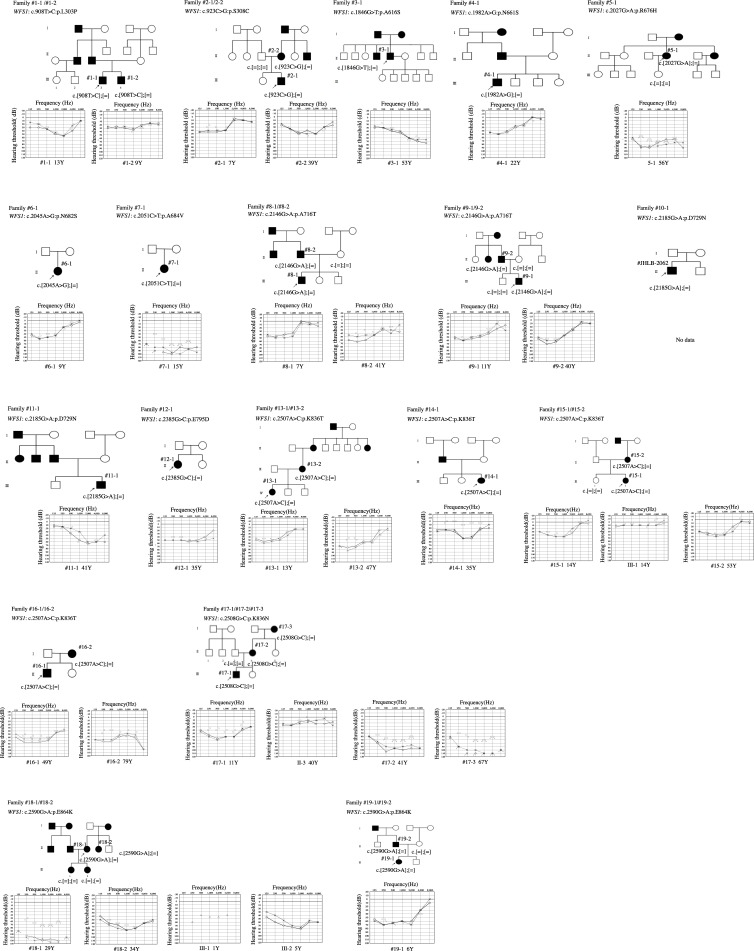
Pedigree and audiograms for each family with a *WFS1* mutation. Arrows show the probands in each family. Genetic findings for each individual tested are noted in the pedigree.

**Fig 2:**
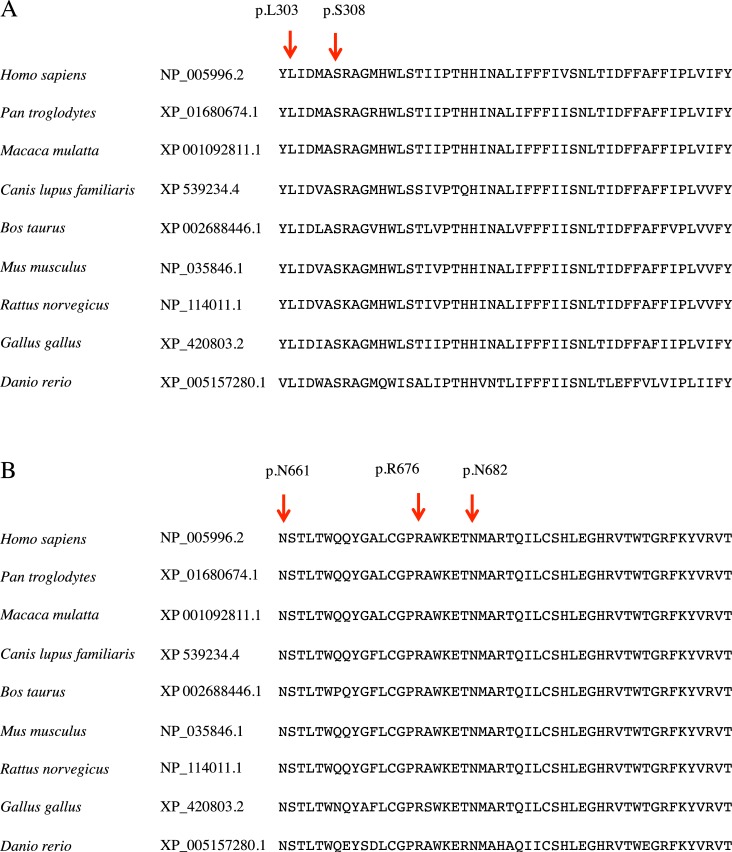
Evolutionary conservation of amino acid residues in the five novel variants. All amino acid residues other than p.S308 are well conserved across vertebrates.

Pedigree and pure-tone audiograms for the 19 probands with *WFS1* variants and their family members are shown in Fig 1. HL in 15 probands exhibited an autosomal dominant inheritance pattern or mitochondrial pattern, whereas HL in the other four probands was sporadic. Three previously reported mutations (p.A684V, p.D729N, and p.E795D) [7, 35, 36] were detected in the sporadic cases. Unfortunately, DNA samples could not be obtained from their parents. However *de novo* changes were thought to be the cause of HL in these sporadic cases. Among the five novel variants, four (p.L303P, p.S308C, p.N661S, and p.R676H) were identified in families with autosomal dominant HL, whereas the other (p.N682S) was detected in a sporadic case. The p.L303P, p.S308C, and p.R676H variants were confirmed to be segregated with HL. Only proband DNA samples were obtained for the remaining two variants (p.N661S, and p.N682S); however, their audiograms showed bilateral low-frequency sensorineural HL, which is the typical configuration in DFNA6/14/38 patients. All five possibly pathogenic variants were predicted to be pathological according to aforementioned prediction software programs (Table 1). All their corresponding amino acid residues are well conserved across vertebrates (Fig 2). Furthermore, these variants were not found in our 333 in-house controls (666 control alleles). Taken together, all five novel variants were considered likely pathogenic variants.

Heterozygous *WFS1* variants were detected in 15 (2.5%) of 602 families with autosomal dominant HL or mitochondrial HL and in four (0.7%) of 559 sporadic cases. No variants were detected in 1018 families with autosomal recessive HL, or 370 families with autosomal recessive or unknown family history. In addition, variants were identified in seven (18.4%) of 38 probands with autosomal dominant low-frequency hereditary HL, and in three (4.5%) of 67 probands with sporadic low-frequency sensorineural HL.

### Clinical audiovestibular features

Table 2 summarizes the clinical characteristics for 30 affected members from 19 families with *WFS1* mutations. [5–7, 34, 36–38]

**Table 2:** Clinical features of affected family members associated with *WFS1* mutations found in this study.

				Hearing loss			Pure-tone audiometry			Other phenotype				
Nucleotide Change	Amino Acid Change	Individual No.	Hereditary	Onset	Progression	Tinnitus	Vertigo/dizziness	Tested age	Audiometric configuration (R/L)	Severity	DM	OA(onset)		Previously reported phenotype (reference)
c.908T>C	p.L303P	1–1	AD	NA	-	-	-	9	LF/LF	Mild	-	-		
		1–2		NA	+	-	-	13	MF/MF	Moderate	-	-		
c.923C>G	p.S308C	2–1	AD	6	-	-	+	7	LF/LF	Moderate	-	-		
		2–2		16	-	-	+	39	MF/MF	Moderate	-	-		
c.1846G>T	p.A616S ※※	3–1	AD	19	-	-	+	53	HF/HF	Moderate	-	-		SNHL* (Liu et al., 2005)
c.1982A>G	p.N661S	4–1	AD	6	-	-	-	22	LF/LF	Moderate	-	-		
c.2027G>A	p.R676H	5–1	AD	6	-	+	-	56	Flat/Flat	Severe	-	-		
c.2045A>G	p.N682S	6–1	Sporadic	4	-	+	-	9	LF/LF	Moderate	-	-		
c.2051C>T	p.A684V	7–1	Sporadic	0	-	-	-	15	Flat/Flat	Profound	-	-		SNHL, OA (Mets et al., 2010)
c.2146G>A	p.A716T	8–1	AD	0	-	-	-	7	LF/LF	Moderate	-	-		LFSNHL, DM (Bespalova et al., 2001)
		8–2		6	-	+	-	41	LF/LF	Moderate	-	-		
		9–1	AD	6	-	-	-	11	LF/LF	Moderate	-	-		
		9–2		15	+	-	-	40	LF/LF	Moderate	-	-		
c.2185G>A	p.D729N	10–1	Sporadic	NA	NA	+	+	29	NA	NA	-	-		LFSNHL, DM (Domènech et al., 2002)
		11–1	AD	28	+	+	+	41	HF/HF	Moderate	-	-		
c.2385G>C	p.E795D	12–1	Sporadic	6	+	+	-	35	LF/Flat	Moderate	-	-		SNHL* (Rohayem et al., 2011)
c.2507A>C	p.K836T	13–1	AD	6	+	+	+	13	LF/LF	Moderate	-	-		MF~LFSNHL (Fujikawa et al., 2010)
		13–2		7	+	+	+	47	LF/LF	Moderate	-	-		
		14–1	AD	6	+	+	-	35	MF/MF	Mild	-	-		
		15–1	AD	6	-	-	-	14	LF/LF	Moderate	-	-		
		15–2		28	+	+	-	53	LF/LF	Moderate	-	-		
		16–1	AD	6	+	+	-	49	LF/LF	Moderate	-	-		
		16–2		NA	+	+	-	79	L and HF/L and HF	Severe	-	-		
c.2508G>C	p.K836N	17–1	AD	5	+	-	-	11	LF/LF	Moderate	-	-		Moderate~severe SNHL, OA (Hogewind et al., 2010)
		17–2		9	+	NA	NA	41	Flat/Flat	Profound	-	-		
		17–3		30	NA	NA	NA	67	Flat/Flat	Profound	NA	NA		
c.2590G>A	p.E864K	18–1	AD	3	+	-	-	29	Flat/Flat	Profound	-	+	(22y.o.)	Moderate SNHL, OA (Eiberg et al., 2006)
		18–2		7	+	-	-	34	MF/MF	Severe	-	+	(unknown)	
		19–1	AD	3	+	-	-	6	LF/LF	Moderate	-	-		
		19–2		NA	NA	-	-	10	NA	NA	-	-		

AD: autosomal dominant. NA: not available. LF: low-frequency sensorineural hearing loss. HF: high-frequency sensorineural hearing loss. MF: middle-frequency sensorineural hearing loss. L and HF: low- and high-frequency sensorineural hearing loss. DM: diabetic mellitus. OA: optic atrophy.

*details unknown

※※p.A616S variant was previously reported as “Pathogenic”. However, this variant was identified over 0.3% of Japanese 1200 control. This is controversial.

The onset age of HL varied widely from 0 to 30 years. Progression of hearing and tinnitus were noticed in 15 (55.5%) of 27 individuals and in 12 (42.9%) of 28 individuals, respectively, while vertigo and/or dizziness was present in seven (25.0%) of 28 individuals.

Pure-tone audiograms could not be obtained for two individuals. In the remaining 28 individuals, audiograms basically showed bilateral symmetrical sensorineural HL (Fig 1). Low-frequency type was the most prevalent audiometric configuration and was recognized in 14 individuals; however, some individuals exhibited flat, middle-frequency (U-shaped), and high-frequency audiograms. In addition, individual 12–1 had a flat audiogram for the left ear and a low-frequency audiogram for the right ear. Individual 16–2 had bilateral low- and high-frequency HL, but the age at audiometric testing was 79 years and the HL at the higher frequencies was thought to be affected by presbycusis.

### Associated symptoms

Three mutations (p.A684V, p.K836N, and p.E864K) previously reported as responsible for Wolfram-like syndrome were detected in four families (Table 2). However, none were found to suffer from diabetes mellitus. Only two affected individuals within the same family individuals (18–1 and 18–2) carrying p.E864K mutation had optic atrophy.

### Haplotype analysis

S2 Table, S3 Table and S4 Table show the haplotype patterns within the 1-Mbp region surrounding the position of the frequent mutations, p.A716T, p.K836T, and p.E864K, respectively. For the p.K836T mutation, in addition to the pedigrees identified in this study, we also analyzed the family with p.K836T reported by Fujikawa et al [34]. in 2010. The pedigree for this case is shown in S1 Fig. The findings suggested that the three mutations occurred on different haplotypes, indicating that the three mutations arose independently in each family and were considered to be mutational hot spots.

## Discussion

In the present study, targeted MPS was carried out in a large series of HL patients, and eight previously reported mutations and additional five novel mutations in *WFS1* were successfully identified in 19 unrelated families. The incidence of *WFS1* variants was 2.5% (15/602) in the presumably autosomal dominant or mitochondrial HL families. This finding shows that *WFS1* mutations are the fourth most frequent cause of autosomal dominant HL in Japan, followed by *KCNQ4* mutations (6.6%) [39], *TECTA* mutations (2.9%) [40], and *POU4F3* mutations (2.7%) [41]. The present study, which enrolled 38 probands with autosomal dominant low-frequency HL, showed a lower *WFS1* mutation detection rate of approximately 20% compared to our previous study (33%) [17].

Mutations causing Wolfram syndrome are spread over the entire coding region in *WFS1*, and are typically inactivating, suggesting that a loss of function causes the disease phenotype [42]. By contrast, although three deletion mutations have been detected in DFNA6/14/38 families, the majority were found to be missense mutations (Table 3) [13–17, 34, 37, 43–56]. The mutations were concentrated in exon 8, and mainly involved amino acid positions in the C-terminal domain (amino acids 652–890). All 13 mutations identified in the present study were missense mutations, and were located in exon 8. Nine of 13 mutations involved amino acid positions in the C-terminal domain (amino acids 652–890).

**Table 3:** Summary of clinical features associated with DFNA6/14/38 from previous studies.

				HL			Pure-tone audiometry				
Nucleotide Change	Exon	Amino Acid Change	Domain	Onset	Progression	Tinnitus	Severity of HL	Audiometric Configuration	Vertigo/dizziness	Vestibular Examination	Reference
c.482G > A	5	R161Q	N-terminal					LF			Barrett et al., 2009
c.511G>A	5	p.D171N	N-terminal	<40	+	+	moderate	LF	-		Gonçalves et al., 2014
c.577A>C	5	p.K193Q	N-terminal	early onset				LF			Cryns et al., 2003
c.799G>A	7	p.D267N	N-terminal								Sloan-Heggen et al., 2016
c.1072G>A	8	p.V358M	TM2								Sloan-Heggen et al., 2015
c.1235T>C	8	p.V412A	TM3								Choi et al., 2013
c.1371G>T	8	p.R457S	–					LF			Smith et al., 2004
c.1554G>A	8	p.M518I	–					LF			Smith et al., 2004
c.1669C> T	8	p.L557F	–					LF			Smith et al., 2004
c.1805C>T	8	p.A602V	TM8					LF			Smith et al., 2004
c.1820C>T	8	p.P607L	TM8								Sloan-Heggen et al., 2016
c.1831C>T	8	p.R611C	–								Sloan-Heggen et al., 2016
c.1846G>T	8	p.A616S	–					LF			Liu et al., 2005
c.1871T>C	8	p.V624A	–					LF			Smith et al., 2004
c.1901A>C	8	p.K634T	TM9	<17		-	moderate	LF			Komatsu et al., 2002
c.1957C>T	8	p.R653C	C-terminal				mild to severe	LF	-		Wei et al., 2014
c.2005T>C	8	p.Y669H	C-terminal	<22	-		moderate	LF			Tsai et al., 2007
c.2021G>A	8	p.G674E	C-terminal	0	+		moderate	LF		Normal	Cryns et al., 2003
c.2021G>T	8	p.G674V	C-terminal	0	+		moderate to severe	LF		Normal	Cryns et al., 2003
c.2033G>T	8	p.W678L	C-terminal					LF			Sivakumaran et al., 2004
c.2036_2038delAGG	8	p.E680del	C-terminal				mild to moderate	LF	-		Wei et al., 2014
c.2053G>C	8	p.R685P	C-terminal	4–	+		moderate to severe	LF		Normal	Bramhall et al., 2008
c.2086C>T	8	p.H696Y	C-terminal	5–28	+	+	mild to profound	LF, Flat	+		Sun et al., 2011
c.2096C>T	8	p.T699M	C-terminal	<25	+		moderate	LF	-	Normal	Bespalova et al., 2001
c.2108G>A	8	p.R703H	C-terminal					LF			Sun et al., 2011
c.2137_2139delGAC	8		C-terminal								Sloan-Heggen et al., 2016
c.2115G>C	8	p.K705N	C-terminal	0	-		moderate	LF			Kunz et al., 2003
c.2141A>T	8	p.N714I	C-terminal								Sloan-Heggen et al., 2016
c.2146G>A	8	p.A716T	C-terminal	<10	+	+	moderate to severe	LF		Normal	Bespalova et al., 2001
c.2209G>A	8	p.E737K	C-terminal								Liu et al., 2005
c.2282C>T	8	p.A761V	C-terminal								Sloan-Heggen et al., 2015
c.2300_2302del	8	p.Idel767	C-terminal	early onset				LF			Cryns et al., 2003
c.2311G>C	8	p.D771H	C-terminal	5–20	-		moderate to severe	LF	-		Gürtler et al., 2005
c.2335G>A	8	p.V779M	C-terminal					LF			Bespalova et al., 2001
c.2389G>A	8	p.D797N	C-terminal	1–17			mild to profound	Flat, HF		Normal	Bai et al., 2014
c.2419A>C	8	p.S807R	C-terminal	early onset				LF			Cryns et al., 2003
c.2486T>C	8	p.L829P	C-terminal	6–32	+	+	moderate	LF	-	Normal	Bespalova et al., 2001
c.2492G>A	8	p.G831D	C-terminal	<20	+	+	moderate	LF	-	Normal	Cryns et al., 2003
c.2507A>C	8	p.K836T	C-terminal	2–10	+	-	moderate	MF, LF	-	Normal	Fujikawa et al., 2010
c.2530G>A	8	p.A844T	C-terminal	<6	-	-	moderate	LF	-	Normal	Noguchi et al., 2005
c.2576G>C	8	p.R859P	C-terminal	5–30	-	-	moderate	LF	-		Gürtler et al., 2005
c.2576G>C	8	p.R859Q	C-terminal	2–45	+	+	moderate	LF	-		Hildebrand et al., 2008
c.2590G>A	8	p.E864K	C-terminal	4	+		moderate to severe	LF	-		Fukuoka et al., 2008
c.2596G>A	8	p.D866N	C-terminal								Liu et al., 2005
c.2603G>A	8	p.R868H	C-terminal								Sloan-Heggen et al., 2016

HL: hearing loss. TM: transmembrane. LF: low-frequency sensorineural hearing loss. HF: high-frequency sensorineural hearing loss. MF: middle-frequency sensorineural hearing loss.

In previous studies, the onset age of HL varied among individuals (Table 3). In the present study, some affected individuals had congenital HL, whereas others exhibited postlingual or late-onset HL, which supported the previous findings. Universal newborn hearing screening has facilitated the detection of congenital HL. However, automated auditory brainstem responses may fail to detect HL in newborns with low-frequency HL, which is the typical audiometric configuration of DFNA6/14/38, as the auditory brainstem response threshold basically reflects the audiometric thresholds at higher frequencies in pure-tone audiograms. Therefore, it is possible that some patients with *WFS1* mutations had congenital HL, even if among patients diagnosed with late-onset HL. The presence of HL progression and/or tinnitus differed by individual, which was consistent with the findings of previous studies. In the present study, two affected individuals possessing Wolfram-like syndrome mutations showed profound HL, whereas the individuals with DFNA6/14/38 showed mild to severe HL. However, a few patients with DFNA6/14/38 were reported to have profound HL [15, 16], and the patients with the same *WFS1* mutations had relatively similar HL.

Although middle-frequency and flat type audiometric configurations were reported in some patients with heterozygous *WFS1* mutations [15, 16, 34], low-frequency type audiograms are typical among DFNA6/14/38 patients. One plausible explanation for these audiometric configuration findings is that *WFS1* mutations were previously screened among patients with low-frequency sensorineural HL. The advantage of this study is that *WFS1* mutations were screened in patients with various types of audiometric configurations. Consequently, although the most prevalent audiometric configuration was low-frequency type, some individuals showed a high-frequency type. Therefore, we should pay attention to the fact that heterozygous *WFS1* mutations can cause high-frequency sensorineural or flat type HL.

Vertigo was reported in several affected members in a Chinese family with the p.H696Y mutation [16]. However, there have been no other studies reporting vertiginous symptoms in DFNA6/14/38 patients. Furthermore, studies on vestibular function, including caloric testing and vestibular evoked myogenic potentials, showed normal function [7, 8, 52]. In this study, seven individuals suffered from vertigo/dizziness; however, none of them underwent vestibular function testing. Further studies are necessary to clarify the relationship between *WFS1* mutations and balance disorders.

Three mutations identified in this study (p.A684V, p.K836N, and p.E864K) were previously reported to cause Wolfram-like syndrome [5–7]. The onset of HL in patients with Wolfram-like syndrome varies from 0 to 15 years of age, and pure-tone audiograms show moderate to profound, symmetric progressive sensorineural HL (Table 4) [5–7, 35, 36]. These patients showed low-frequency, flat, or middle-frequency audiometric configurations. The Wolfram-like syndrome phenotypes were almost identical to those of DFNA6/14/38. In addition, all reported patients had optic atrophy, whereas only some patients exhibited diabetes mellitus [5–7]. In this study, HL started from 0 to 30 years of age, and was moderate to profound in severity. Audiometric configurations included flat, low-frequency, and middle-frequency types. These findings were similar to those of previous studies on Wolfram-like syndrome. However, no individual suffered from diabetes mellitus, and only two individuals had optic atrophy. It is necessary to follow patients due to the possibility of late-onset diabetes mellitus and optic atrophy [5].

**Table 4:** Summary of clinical features associated with Wolfram-like syndrome from previous studies.

				HL			Pure-tone audiometry				Other Phenotypes		
Nucleotide Change	Exon	Amino Acid Change	Domain	Onset	Progression	Tinnitus	Severity of HL	Audiometric Configuration	Vertigo/dizziness	Vestibular Examination	DM	OA	Reference
c.2051C>T	8	p.A684V	C-terminal	early childhood	+		severe to profound	Flat			+	+	Rendtorff et al., 2011
c.2185G>A	8	p.D729N	C-terminal								+		Domenech et al., 2002
c.2269C>A	8	p.D757I	C-terminal								+		Domenech et al., 2002
c.2338G>C	8	p.G780S	C-terminal	congenital	–		profound				–	+	Rendtorff et al., 2011
c.2385G>C	8	p.E795D	C-terminal	1~20							+	+	Rohayen et al., 2011
c.2389G>T	8	p.D797Y,	C-terminal	3<4	+		severe to profound	Flat			–	+	Rendtorff et al., 2011
c.2508G>C	8	p.K836N	C-terminal	8<14	+		severe	Flat		Normal	–	+	Hogewind et al., 2010
c.2590G>A	8	p.E864K	C-terminal	childhood	+		moderate to severe	LFSNHL, Flat			Partially	+	Eiberg et al., 2006
c.2611G>A	8	p.V871M	C-terminal								+		Domenech et al., 2002

Haplotype analysis of p.A716T, p.K836T, and p.E864K did not show the same haplotype among the families with the same mutation. In addition, p.A716T and p.E864K, which were detected in our Japanese population, have also been reported in the other ethnic populations. These findings suggested that the three mutations occurred in mutational hot spots. Interestingly, four variants in *WFS1* were identified among our sporadic cases; however, three of the four variants (p.N682S, p.A684V, p.D729N, and p.E795D) have been previously reported in non-Asian populations. Therefore, these variants might occur as *de novo* changes at the mutational hot spots.

In summary, this MPS-based study successfully identified eight previously reported mutations and five novel variants, and estimated the incidence of *WFS1* variants to be 2.5% in Japanese families with presumably autosomal dominant or mitochondrial HL. This exhaustive mutational study of a large series of HL patients provided valuable new insights, particularly with regard to the audiometric configurations and vestibular symptoms in DFNA6/14/38 or Wolfram-like syndrome patients. We found that some variants can occur as a *de novo* change at the mutational hot spots in *WFS1*, resulting in an audiovestibular phenotype.

## Supporting information

S1 Table The 68 genes reported to cause hearing loss. (PDF)

S2 Table Haplotype patterns of two families with p.A716T. (PDF)

S3 Table Haplotype patterns of five families with p.K836T. (PDF)

S4 Table Haplotype patterns of two families with p.E864K. (PDF)

S1 Fig Pedigree of a proband with *WFS1*–associated hearing loss with a p.K836T mutation reported in Fuijkawa et al, 2010. (PDF)
