# Supplementary material for: WFS1 mutation screening in a large series of Japanese hearing loss patients: Massively parallel DNA sequencing-based analysis
Source: PLoS One. 2018 Mar 12;13(3):e0193359. doi: 10.1371/journal.pone.0193359 (PMC5846739; doi:10.1371/journal.pone.0193359)
Supplement: S2 Table — (PDF) [file pone.0193359.s002.pdf]

Supplementary Table S2;Haplotype patterns of two c.2146G>A families.

Distance from the WFS1 mutation(bp)	Fm8						Fm9						Marker
	Son(#8-1)		Fa(#8-2)		Mo(Ⅱ-3)		Son(#9-1)		Fa(#9-2)		Mo(Ⅱ-4)		
	Affected		Affected		Unaffected		Affected		Affected		Unaffected		
	A	U	A	U			A	U	A	U			
1075511	T	C	T	C	C	C	C	C	C	T	C	C	rs985222
1025873	T	G	T	G	G	G	T	T	T	G	T	T	rs16836949
959210	T	C	T	C	C	T	T	T	T	C	C	T	rs6817527
891696	C	C	C	T	C	T	C	T	C	T	C	T	rs195112
838493	C	C	C	C	C	C	C	C	C	T	C	C	rs16837322
775084	T	T	T	T	T	T	T	T	T	T	T	T	rs10488937
761891	C	C	C	C	C	C	C	C	C	C	C	C	rs10937648
645131	T	C	T	T	C	C	C	T	C	C	C	T	rs4688945
562995	C	C	C	T	C	C	C	T	C	C	T	T	rs10440166
478798	A/G	A/G	A/G	A/G	A	G	A/G	A/G	A/G	A/G	A	G	rs3774883
442363	A	A	A	G	A	A	G	G	G	G	A	G	rs13130069
380637	T	T	T	C	T	T	T	T	T	T	T	T	rs1075684
323287	C/T	C/T	C/T	C/T	C	T	C/T	C/T	C/T	C/T	C	T	rs6821688
219852	A	G	A	G	G	G	A	G	A	G	G	G	rs4689334
161787	T	C	T	C	C	T	T	T	T	C	C	T	rs10017549
93846	C	C	C	C	C	C	C	C	C	C	C	C	rs4689360
48140	C	C	C	C	C	C	C	C	C	C	C	C	rs4689382
8948	G	G	G	G	G	G	T	G	T	G	G	G	rs12511742
2041	A	A	A	A	A	A	A	A	A	A	A	A	rs734312
0	-		-		-		-		-		-		c.2146G>A
38842	A	G	A	G	A	G	A	A	A	A	A	A	rs4689411
70671	A	A	A	A	A	A	A	G	A	G	A	G	rs12651287
136020	G	A	G	G	A	G	G	A	G	A	A	G	rs17722973
217881	G	T	G	G	G	T	T	T	T	G	T	T	rs4075006
265404	G	G	G	G	A	G	G	G	G	G	G	G	rs10937743
344224	T	T	T	T	T	T	T	C	T	C	C	T	rs4689024
428264	A/G	A/G	A/G	A/G	A	G	A/G	A/G	A/G	A/G	A	G	rs4420983
450464	C	C	C	T	C	C	T	T	T	T	T	T	rs10032820
497421	C	C	C	G	C	G	C	C	C	G	C	C	rs879329
588286	G	A	G	A	A	G	G	G	G	G	G	G	rs3901368
636496	C	C	C	C	C	C	C	C	C	C	C	C	rs11723719
706463	C	G	C	G	C	G	C	G	C	G	G	G	rs2301820
746787	C/T	C/T	C/T	C/T	C	T	C/T	C/T	C/T	C/T	C	T	rs870660
788439	G	A	G	A	A	A	A	A	A	G	A	G	rs11734660
846313	A	A	A	A	A	A	G	G	G	A	A	G	rs10021205
859370	C	C	C	C	C	C	C	C	C	C	C	C	rs9291130
909538	A	G	A	G	A	G	A	G	A	A	A	G	rs11937057

Fm(n), Family number(n); Mo, Mother; Fa, Father; A, Affected allele; U, Unaffected allele.

Putative haplotype for affected allele

Different SNPs among 2 affected families
