# Supplementary material for: WFS1 mutation screening in a large series of Japanese hearing loss patients: Massively parallel DNA sequencing-based analysis
Source: PLoS One. 2018 Mar 12;13(3):e0193359. doi: 10.1371/journal.pone.0193359 (PMC5846739; doi:10.1371/journal.pone.0193359)
Supplement: S3 Table — (PDF) [file pone.0193359.s003.pdf]

Supplementary Table S3;Haplotype patterns of five c.2507A>C families.

Distance from the WFS1 mutation(bp)	Fm13				Fm14		Fm15				Fm16				Fm(Fujika		
	Dau(#13-1)		Mo(#13-2)		Dau(#14-1)		Dau(#15-1)		Mo(#15-2)		Son(#16-1)		Mo(#16-2)		Ⅳ-4		Ⅲ
	Affected		Affected		Affected		Affected		Affected		Affected		Affected		Affected		Unaff
	A	U	A	U	A	U	A	U	A	U	A	U	A	U	A	U	
1075872	C	T	C	C	C	T	C	C	C	C	C	C	C	C	C	C	C
1026234	G/T	G/T	G/T	G/T	G/T	G/T	G/T	G/T	G/T	G/T	T	G	T	T	G	G	G
959571	C	T	C	C	C	T	C	T	C	T	C	T	C	T	C	T	C
892057	T	C	T	T	C/T	C/T	T	T	T	C	T	C	T	T	C	T	C
838854	C	T	C	C	C	T	C	C	C	T	C	T	C	T	C	C	C
775445	T	A	T	A	T	T	T	T	T	T	T	T	T	T	T	T	A
762252	C	A	C	C	C	C	C	C	C	C	C	C	C	C	C	C	C
645492	C	C	C	C	C	T	C	C	C	T	C	C	C	C	C	C	C
563356	T	C	T	T	C	C	T	T	T	T	T	C	T	T	T	C	C
479159	A	A	A	G	A	G	A	A	A	A	A	G	A	A	A	A	A
442724	A	A	A	A	A	G	A	A	A	A	A	A	A	A	A	A	A
320948	C/T	C/T	C/T	C/T	C	C	C/T		C/T		C/T		C/T		T	T	T
220213	G	G	G	G	G	A	G	G	G	G	G	G	G	G	G	G	A
162148	T	T	T	T	T	T	T	C	T	T	T	T	T	C	T	C	C
94207	C	C	C	C	C	C	C	C	C	C	C	C	C	C	C	C	A
48501	C	C	C	C	C	C	C	C	C	C	C	C	C	C	C	C	C
9309	G	G	G	G	G	G	G	G	G	T	G	G	G	G	G	G	G
2402	A	A	A	A	A	A	A	A	A	A	A	A	A	A	A	A	A
0	-		-		-		-		-		-		-		-		-
38481	A	A	A	A	A	A	A	G	A	G	A	A	A	A	A	A	A
73310	A	A	A	G	A	A	A	G	A	G	A	A	A	A	A	A	A
135659	A	G	A	G	A	G	A	A	A	G	A	A	A	G	A	G	A
217520	G	G	G	T	G	T	G	G	G	T	G	G	G	G	G	G	G
265043	A	G	A	G	A	A	A	G	A	A	A	A	A	G	A	A	A
343863	T	C	T	T	T	T	T	T	T	C	T	T	T	T	T	C	C
427903	A	G	A	G	A	G	A	G	A	G	A	G	A	G	A	A	A
450103	C	C	C	C	C	C	C	C	C	C	C	C	C	C	C	C	C
497060	C	C	C	C	C	C	C	C	C	C	C	G	C	G	C	C	C
586925	G	G	G	A	G	G	G	G	G	A	G	A	G	A	G	G	A
636135	C	C	C	C	C	C	C	C	C	C	C	C	C	C	T	C	C
706102	C	C	C	C	C	G	C	C	C	C	C	C	C	C	C	G	C
746426	C	T	C	T	C	T	C	T	C	T	C	T	C	T	C	T	C
788078	A	A	A	A	A	A	A	A	A	A	A	G	A	A	A	A	A
845952	A	A	A	A	A	G	A	A	A	A	A	A	A	A	A	A	A
859009	C	T	C	T	C	C	C	C	C	C	C	C	C	T	C	C	C
909177	G	A	G	A	G	G	G	A	G	G	G	G	G	A	G	A	A

Fm(n), Family number(n); Mo, Mother; Fa, Father; Dau, Daughter; A, Affected allele; U, Unaffected allele.

Putative haplotype for affected allele

Different SNPs among 5 affected families

awa)

-8	Ⅲ-9		Ⅱ-5		Ⅲ-2		Ⅲ-3		Ⅳ-2		Ⅳ-7		Ⅳ-8		Ⅲ-10		Ⅲ-11		
ected	Affected		Affected		Affected		Unaffected		Unaffected		Affected		Affected		Affected		Unaffected		Marker
	A	U	A	U	A	U					A	U	A	U	A	U			
C	C	C	C	C	C	C	C	C	C	T	C	C	C	C	C	C	C	C	rs985222
T	G	T	G	G	G	T	G	T	T	T	G	T	G	T	G	T	T	T	rs16836949
T	C	T	C	C	C	T	C	T	T	T	C	C	C	T	C	T	C	T	rs6817527
T	C	T	C	T	C	C	C	T	C	C	C	T	C	T	C	T	C	T	rs195112
T	C	C	C	T	C	T	C	T	C	T	C	T	C	T	C	C	C	T	rs16837322
T	T	T	T	T	T	T	A	T	A	T	T	T	T	T	T	T	T	T	rs10488937
C	C	C	C	C	C	C	C	C	C	C	C	C	C	C	C	C	C	C	rs10937648
C	C	T	C	C	C	C	C	C	C	T	C	C	C	T	C	T	C	T	rs4688945
T	T	C	T	C	T	T	T	T	T	T	T	C	T	C	T	C	C	T	rs10440166
A	A	A	A	A	A	A	A	A	A	A	A	G	A	G	A	A	A	G	rs3774883
A	A	A	A	A	A	A	A	A	A	A	A	A	A	G	A	G	A	G	rs13130069
T	T	T	T	T	T	T	T	T	T	T	T	T	T	T	T	T	C	T	rs6821688
G	G	G	G	G	G	G	G	G	A	G	G	G	G	G	G	G	G	G	rs4689334
T	T	T	T	T	T	T	T	T	T	T	T	C	T	C	T	T	C	C	rs10017549
C	C	C	C	A	C	C	A	C	C	C	C	C	C	C	C	C	C	C	rs4689360
C	C	C	C	C	C	C	C	C	C	C	C	C	C	C	C	C	C	T	rs4689382
T	G	G	G	G	G	G	G	G	G	G	G	G	G	G	G	G	G	G	rs12511742
A	A	A	A	G	A	A	A	G	A	A	A	A	A	A	A	A	A	A	rs734312
	-		-		-		-		-		-		-		-		-		c.2507A>C
A	A	G	A	G	A	G	G	G	A	G	A	A	A	A	A	A	A	G	rs4689411
A	A	A	A	A	A	A	A	A	A	G	A	A	A	A	A	G	A	G	rs12651287
G	A	A	A	A	A	A	A	G	A	G	A	A	A	G	A	G	A	G	rs17722973
G	G	G	G	G	G	G	G	G	G	G	G	G	G	G	G	G	G	G	rs4075006
G	A	G	A	G	A	G	G	G	G	G	A	G	A	G	A	G	G	G	rs10937743
T	T	T	T	T	T	C	C	T	C	T	T	T	T	T	T	T	C	T	rs4689024
A	A	G	A	G	A	A	A	G	A	G	A	A	A	A	A	G	A	A	rs4420983
T	C	T	C	T	C	C	C	T	C	C	C	C	C	C	C	C	C	C	rs10032820
G	C	G	C	C	C	G	C	G	G	G	C	C	C	G	C	C	C	G	rs879329
G	G	A	G	A	G	A	A	G	A	A	G	A	G	A	G	G	A	G	rs3901368
C	T	T	T	C	T	C	C	T	C	C	T	C	T	C	T	T	C	T	rs11723719
G	C	C	C	C	C	C	C	C	C	G	C	C	C	C	C	C	C	C	rs2301820
T	C	T	C	C	C	T	C	T	C	T	C	T	C	T	C	T	T	T	rs870660
A	A	A	A	G	A	A	A	G	A	G	A	A	A	A	A	G	A	A	rs11734660
A	A	A	A	A	A	G	A	G	A	G	A	A	A	G	A	A	A	G	rs10021205
T	C	C	C	T	C	T	T	T	C	T	C	C	C	C	C	C	C	T	rs9291130
G	G	G	G	A	G	G	A	G	A	G	G	A	G	A	G	A	A	A	rs11937057
