# Supplementary material for: WFS1 mutation screening in a large series of Japanese hearing loss patients: Massively parallel DNA sequencing-based analysis
Source: PLoS One. 2018 Mar 12;13(3):e0193359. doi: 10.1371/journal.pone.0193359 (PMC5846739; doi:10.1371/journal.pone.0193359)
Supplement: S4 Table — (PDF) [file pone.0193359.s004.pdf]

Supplementary Table S4;Haplotype patterns of two c.2590G>A families.

Distance from the WFS1 mutation(bp)	Fm18				Fm19						Marker
	Mo(#18-1)		Au(#18-2)		Dau(#19-1)		Fa(#19-2)		Mo(Ⅱ -3)		
	Affected		Affected		Affected		Affected		Unaffected		
	A	U	A	U	A	U	A	U			Marker
1075955	C	C	C	C	C	T	C	T	C	T	rs16836949
1026317	T	G	T	T	T	G	T	G	G	T	rs985222
959654	T	C	T	T	T	C	T	C	C	T	rs6817527
892140	C/T	C/T	C/T	C/T	C/T	C/T	C/T	C/T	C	T	rs195112
838937	C	C	C	C	C	C	C	T	C	C	rs16837322
775528	T	T	T	T	A	T	A	A	A	T	rs10488937
762335	C	A	C	C	A	A	A	A	A	C	rs10937648
645575	C	C	C	C	C	C	C	C	C	C	rs4688945
563439	T	C	T	C	T	T	T	C	T	T	rs10440166
479242	A	A	A	A	A	G	A	G	A	G	rs3774883
442807	A	A	A	A	A	A	A	A	A	A	rs3774883
381081	T	T	T	T	T	T	T	T	T	T	rs13130069
323731	T	C	T	C	T	T	T	T	T	T	rs6821688
220296	G	G	G	G	G	A	G	G	G	A	rs4689334
162231	T	T	T	T	T	T	T	C	T	T	rs10017549
94290	C	C	C	C	C	C	C	C	C	C	rs4689360
48584	C	C	C	C	C	C	C	C	C	G	rs4689382
9392	G	G	G	G	G	T	G	T	G	T	rs12511742
2485	A	A	A	A	A	A	A	A	A	A	rs734312
0	-		-		-		-		-		c.2590G>A
38398	A	A	A	A	G	A	G	A	A	A	rs4689411
70227	G	A	G	A	G	A	G	A	A	A	rs12651287
135576	G	A	G	A	G	G	G	G	A	G	rs17722973
217437	G	T	G	G	T	T	T	G	G	T	rs4075006
264960	G	G	G	G	G	G	G	G	A	G	rs10937743
343780	T	T	T	T	T	T	T	C	C	T	rs4689024
427820	A	A	A	A	G	G	G	G	G	G	rs4420983
450020	C	C	C	C	C	C	C	C	C	C	rs10032820
496977	G	G	G	C	C	C	C	C	C	C	rs879329
587842	A	G	A	A	A	G	A	G	A	G	rs3901368
636052	C	C	C	C	C	C	C	C	C	C	rs11723719
706019	G	G	G	C	C	C	C	G	C	G	rs2301820
746343	C	C	C	T	C	T	C	T	C	T	rs870660
787995	A	G	A	G	A	A	A	A	A	A	rs11734660
845869	A	A	A	A	A	A	A	A	A	A	rs10021205
858926	C	C	C	C	C	T	C	C	C	T	rs9291130
909094	G	G	G	G	G	G	G	A	G	G	rs11937057

Fm(n), Family number(n); Mo, Mother; Fa, Father; Dau, Daughter; Au, Aunt; A, Affected allele; U, Unaffected allele.

Putative haplotype for affected allele

Different SNPs among 2 affected families
