# Supplementary figures and images for: WFS1 mutation screening in a large series of Japanese hearing loss patients: Massively parallel DNA sequencing-based analysis
Source: PLoS One. 2018 Mar 12;13(3):e0193359. doi: 10.1371/journal.pone.0193359 (PMC5846739; doi:10.1371/journal.pone.0193359)

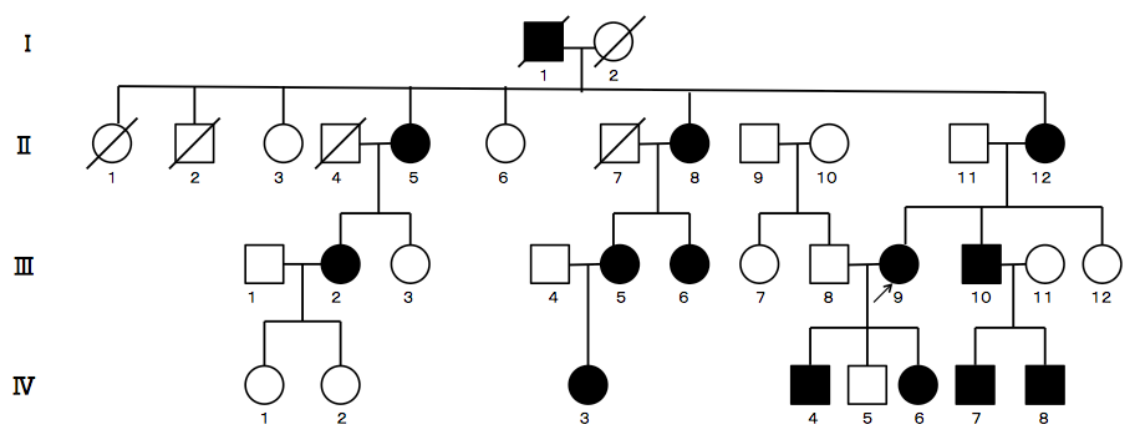

Supplement: S1 Fig — (PDF) [file pone.0193359.s005.pdf]
